# Platelet-Rich Plasma Enhances Adhesion and Short-Term Retention of Bone Marrow-Derived Mesenchymal Stromal Cells to Articular Cartilage

**DOI:** 10.3390/cells15111024

**Published:** 2026-06-02

**Authors:** Sung Yong Ahn, Chris Hyunchul Jo

**Affiliations:** 1 Department of Physiology, Ajou University School of Medicine, Suwon 16499, Republic of Korea; 2 Department of Orthopedic Surgery, SMG-SNU Boramae Medical Center, Seoul National University College of Medicine, Seoul 07061, Republic of Korea

**Keywords:** mesenchymal stromal cells, cartilage regeneration, osteochondral defect, extracellular matrix, cell engraftment, regenerative medicine, osteoarthritis

## Abstract

Mesenchymal stromal cell (MSC) adhesion and retention at sites of cartilage degeneration are critical for improving cartilage repair. This study investigated whether platelet-rich plasma (PRP) enhances the adhesion and short-term retention of bone marrow-derived MSCs (BM-MSCs) and chondrocytes under in vitro and ex vivo conditions. BM-MSCs and chondrocytes were treated with PRP or pretreated with PRP for 10 or 30 min, and cell adhesion to collagen-coated surfaces was evaluated using a cell viability assay. Ex vivo adhesion and short-term retention of BM-MSCs on osteochondral discs with varying lesion severity were assessed by fluorescence imaging analysis. PRP significantly enhanced the adhesion of both BM-MSCs and chondrocytes in a time-dependent manner, with the 30 min PRP pretreatment group showing the greatest effect. BM-MSC attachment in the 30 min PRP pretreatment group was significantly higher than that in the untreated control group after 30 min of incubation (*p* < 0.001), whereas chondrocyte attachment was also significantly increased following PRP pretreatment. In addition, PRP pretreatment significantly enhanced BM-MSC attachment compared with PRP treatment alone at 20 and 30 min of incubation (both *p* < 0.001). In ex vivo experiments, adhesion and short-term retention increased significantly with increasing lesion severity from G1 to G3 (*p* < 0.05 and *p* < 0.01, respectively). In G2 and G3 lesions, PRP pretreatment for 30 min significantly enhanced BM-MSC adhesion and short-term retention compared with the control group (both *p* < 0.01). These findings suggest that PRP may improve the early adhesion and retention of MSCs on damaged cartilage and support the potential use of PRP as a biological adjunct for MSC-based cartilage repair strategies.

## 1. Introduction

Cell therapy refers to the administration of autologous or allogeneic cellular components for therapeutic purposes and is widely applied in regenerative medicine, immunotherapy, and cancer treatment [1]. These cellular therapies are broadly categorized as advanced therapy medicinal products (ATMPs), which include somatic cell therapies and tissue-engineered products regulated under established regulatory frameworks. Among stem cell–based approaches, mesenchymal stromal cells (MSCs) have attracted considerable attention because of their regenerative and immunomodulatory properties [2]. However, the therapeutic efficacy of transplanted MSCs is often limited by poor homing, engraftment, and survival within injured tissues [3]. Anoikis is a form of programmed cell death that is triggered in anchorage-dependent cells when they lose attachment to the surrounding extracellular matrix (ECM) [4]. In addition, culture-expanded MSCs frequently exhibit reduced expression of chemokine receptors associated with tissue homing, resulting in lower engraftment efficiency [5,6]. Therefore, improving MSC adhesion and retention within damaged tissues may improve the therapeutic efficacy of MSC-based therapies. Integrins mediate MSC adhesion to ECM proteins such as collagen and fibronectin and play critical roles in cell migration, survival, and cartilage regeneration [7,8,9].

Platelets contain various bioactive substances within their granules, including inflammatory factors, growth factors, cytokines, chemokines, adhesion proteins, proteases, and antiproteases associated with tissue repair or regeneration [10,11,12]. Platelet-rich plasma (PRP) is a platelet concentrate comprising at least 1000 × 10^3^ platelets/μL, which is about three- to fivefold greater than in whole blood [13]. PRP contains various bioactive substances within platelet granules at concentrations higher than physiological levels [14]. Moreover, PRP has the capacity to restore or regenerate damaged vascular tissues and is therefore used in the treatment of conditions such as arthritis, chronic lower back pain, chronic pelvic pain, and ligament injuries of the shoulder or knee, functioning as a stimulus to enhance tissue repair in prolotherapy and skin regeneration. PRP promotes tissue healing and regeneration in various musculoskeletal tissues, including bone, cartilage, tendons, ligaments, and muscles [15,16,17,18,19,20]. Recent studies have further highlighted the therapeutic potential of PRP in osteoarthritis and regenerative musculoskeletal medicine [21,22]. However, the mechanisms by which PRP enhances tissue repair and MSC engraftment remain incompletely understood [23]. Previous studies and related intellectual property from our group have suggested that PRP may enhance the engraftment, attachment, and homing properties of transplanted cells, including MSCs [24]. However, direct experimental evidence regarding the effects of PRP on the early adhesion and short-term retention of MSCs on damaged articular cartilage remains limited.

In articular cartilage, surface molecules such as aggrecan, decorin, and biglycan interfere with cell engraftment, resulting in poor cell adherence [25,26]. Previous studies demonstrated that stem cells injected into the joint space were primarily distributed within the synovium, meniscus, and fat pad, with minimal attachment to articular cartilage lesions [27,28]. These findings suggest that insufficient engraftment and retention of transplanted MSCs remain major limitations in stem cell-based cartilage repair. Improving the adhesion and retention of MSCs at cartilage lesions is considered an important strategy for enhancing the therapeutic efficacy of MSC-based treatment for osteoarthritis. However, despite the widespread combined use of PRP and MSCs for osteoarthritis treatment, whether PRP directly enhances the adhesion and short-term retention of MSCs on damaged articular cartilage remains unclear. In particular, the early adhesion-related effects of PRP on MSC engraftment to cartilage lesions have not been sufficiently investigated. Therefore, this study aimed to investigate whether PRP enhances the adhesion and short-term retention of MSCs on injured articular cartilage.

## 2. Materials and Methods

### 2.1. Mesenchymal Stromal Cells (MSCs)

Human mesenchymal stromal cells (MSCs) were obtained from the bone marrow of patients and healthy individuals using a standard procedure. Ficoll-Paque^TM^ PREMIUM was used to isolate monocytes via centrifugation. The cells were then washed twice and resuspended in low glucose Dulbecco’s modified Eagle’s medium (DMEM-LG; HyClone, Thermo Fisher Scientific Inc., Waltham, MA, USA) supplemented with 10% bovine serum albumin (HyClone, Thermo Fisher Scientific Inc.) supplemented with 100 U/mL penicillin and 100 μg/mL streptomycin (HyClone, Thermo Fisher Scientific Inc.), seeded at a concentration of 1 × 10^6^ cells/cm^2^, and incubated at 37 °C in a 5% CO_2_ incubator with humidity. The medium was changed every 1–2 days after the start of the culture, and the medium was then changed every 3 days. When cells reached 80% confluence, they were passaged at a split ratio of 1:3. In all experiments, MSCs were used at a passage <10. The cells used in this study were previously characterized by flow cytometric analysis of MSC-associated surface markers (CD73, CD90, and CD105 positive; CD11a, CD34, and CD45 negative) and trilineage differentiation potential, including adipogenic, osteogenic, and chondrogenic differentiation, as described in our previous study [29].

### 2.2. Chondrocytes

Cartilage tissue was washed with phosphate-buffered saline (PBS) without calcium and magnesium, and minced into small pieces for chondrocyte isolation. High-glucose DMEM (Life Technologies, Rockville, MD, USA), containing 100 U/mL penicillin, 100 mg/mL streptomycin, 0.25 mg/mL amphotericin B (Life Technologies), and 0.6% collagenase (Sigma-Aldrich, St. Louis, MO, USA), was added for 6 h at 37 °C to release chondrocytes. A nylon mesh with a 100 nm pore size (BD, Franklin Lakes, NJ, USA) was used to remove any chondrocytes that were not minced. The filtered material was then centrifuged to obtain the chondrocytes, which were then washed twice. The cells were then resuspended in DMEM containing 10% fetal bovine serum (FBS, Life Technologies) supplemented with 25 mg/mL L-ascorbic acid (Sigma-Aldrich), 100 U/mL penicillin, and 100 μg/mL streptomycin (HyClone, Thermo Fisher Scientific Inc.), seeded onto a 10 mm culture dish, and incubated at 37 °C in a 5% CO_2_ incubator with humidity for 7 days. The medium was changed at 1–2 d after the start of the culture, and later every 3 days. When cells reached 80% confluence, they were passaged at a split ratio of 1:3. The cells were treated with trypsin-EDTA solution (0.25% trypsin, 0.53 mM EDTA; Life Technologies) for 5 min to detach from the dish, washed twice with culture medium, and resuspended in PBS or PRP. Cells up to passage two to five were used in the experiment.

### 2.3. Preparation of PRP

Platelet-rich plasma (PRP; *n* = 3) was prepared from three independent healthy donors using a plateletpheresis system equipped with a leukoreduction set (COBE Spectra LRS Turbo, Caridian BCT, Lakewood, CO, USA) and a standard collection program consisting of a 90 min double-needle procedure [30]. PRP samples from different donors were not pooled, and each donor-derived PRP preparation was used in independent biological experiments. Saline and anticoagulant acid citrate dextrose (ACDA) were prepared as anticoagulant agents according to the manufacturer’s instructions. The platelet concentration of PRP was adjusted to 1000 × 10^3^ platelets/μL for the experiments [14]. The PRP preparation protocol used in this study was based on our previously published leukocyte-poor PRP (LP-PRP) system generated by plateletpheresis, which demonstrated standardized platelet enrichment with minimal red and white blood cell contamination [30]. To activate PRP, a 10% calcium gluconic acid salt comprising 166.7 IU/mL of thrombin (Reyon Pharmaceutical, Seoul, Republic of Korea) was added to the PRP at a 1:10 vol/vol ratio. The control group was treated with saline alone. All procedures involving human samples were conducted in accordance with the Declaration of Helsinki and approved by the Institutional Review Board of SMG-SNU Boramae Medical Center, Seoul National University College of Medicine, Seoul, Republic of Korea (No. 30-2019-124; Development of the 2nd Generation Bone Marrow Stimulation Strategy via Proliferation & Recruitment of Endogenous Stem Cells for the Treatment of Cartilage and Tendon Defects; approved on 5 December 2019). Written informed consent was obtained from all participants prior to sample collection.

### 2.4. Characterization of PRP

Complete blood (platelets, red blood cell, and white blood cell) counts were measured using a fully automated analyzer (XE-2100, Sysmex Corp, Kobe, Japan). The characteristics of PRP have been reported in our previous studies [30]. In this study, the whole-blood platelet count increased from 261.26 ± 58.68 to 1595.74 ± 454.08 × 10^3^ platelets/μL after plateletpheresis. The red blood cell count decreased from 4.29 ± 0.42 × 10^6^ cells/μL before plateletpheresis to 0.21 ± 0.09 × 10^6^ cells/μL after plateletpheresis. The white blood cell count decreased from 6.10 ± 1.96 × 10^3^ cells/μL before plateletpheresis to 0.38 ± 1.15 × 10^3^ cells/μL after plateletpheresis. After adjustment to a target concentration of 1000 × 10^3^ platelets/μL using saline, the mean actual platelet count was 900.86 ± 78.06 × 10^3^ platelets/μL. To minimize donor-to-donor variability, all PRP preparations were adjusted to the same target platelet concentration prior to use in experiments.

### 2.5. In Vitro Cell Adhesion Assay

BM-MSCs and chondrocytes were suspended in saline (control group) or treated with PRP before seeding onto plates. The experimental groups were as follows: Control (suspended in saline); PRP (suspended in PRP); pretreated with PRP for 10 min (cells were suspended in PRP for 10 min before the transplant); pretreated with PRP for 30 min (cells were suspended in PRP for 30 min before the transplant). A 30 min pretreatment period was selected to evaluate early adhesion-related responses. Cells were seeded into each well of a 96-well plate and incubated at 37 °C in a 5% CO_2_ incubator for 2.5, 5, 10, 15, 20, and 30 min. Culture plates were coated with 10 µg/mL type I collagen (Corning Incorporated, Corning, NY, USA) and incubated overnight at 4 °C. Nonspecific binding was blocked with 1% bovine serum albumin (BSA; Sigma-Aldrich) in DPBS for 90 min at 37 °C. BM-MSCs and chondrocytes (1 × 10^5^ cells/cm^2^) were seeded onto precoated plates and incubated at 37 °C for 30 min. Each well was washed twice with DPBS to remove unattached cells after incubation. The cells were viewed under an inverted research microscope (Leica DM IL microscope, Wetzlar, Germany). The number of adherent cells was measured using a cell viability assay kit (EZ-CYTOX, Cell Viability Assay Kit; Daeillab, Seoul, Republic of Korea), while the optical density of the microplate wells was measured using a microplate reader (SpectraMax Plus384; Molecular Devices, Sunnyvale, CA, USA).

### 2.6. Osteochondral Disc Preparation

**Figure 1:**
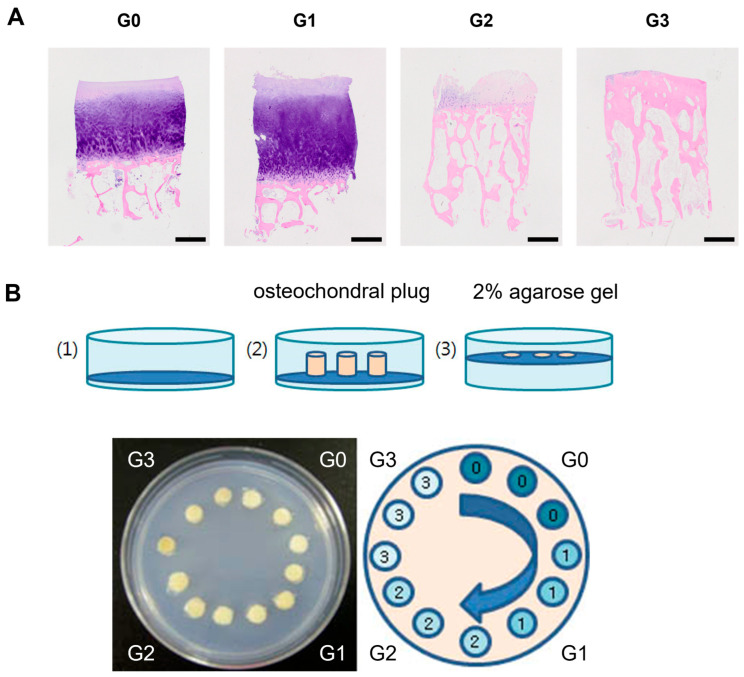
Histological differences in osteochondral plugs according to injury severity (**A**) Hematoxylin and eosin (H&E) staining. G0: normal; G1: damage or lesion progression of cartilage thickness <50%; G2: damage or lesion progression of cartilage thickness >50%; G3: lesions in which the damage or lesion extends to the subchondral bone, exposing the subchondral osseous lamina (original magnification, 12.5×; scale bar = 1 mm). (**B**) Schematic drawing of osteochondral plug preparation and arrangement in a 2% agarose gel. Grade zero plugs were placed on the upper right side, grade one plugs were placed on the lower right side, grade two plugs were placed on the lower left side, and grade three plugs were placed on the upper left side in a clockwise direction.

The osteochondral block was prepared using a method commonly used in the field, with minor modifications. The proximal tibial fragment used for the ex vivo experiments was derived from a single patient who underwent knee joint replacement surgery. The proximal tibia was evaluated and graded according to the criteria established by the International Cartilage Repair Society (ICRS) [31]. The grading system for injuries was based on the injury severity, with the following categories: zero for normal; one for a reduction in cartilage thickness at the lesion of <50%; two for a reduction in cartilage thickness at the lesion of >50%; and three for exposure of the subchondral osseous lamina at the lesion (Figure 1A).

Osteochondral plugs were prepared in a cylindrical shape (diameter: 4.0 mm, height: 4.0 mm) using a Trephine burr. The plugs were stored at −80 °C and thawed at 25 °C in DPBS for 1 h before use. The variability caused by visual grading was overcome by grading multiple times. The dissolved agarose gel was carefully poured to cover the bottom of a 35 mm culture dish. Osteochondral plugs were placed in 35 mm culture dishes and embedded in 2% agarose gel, leaving the cartilage surface exposed upward. Grade zero plugs were placed on the upper right side, grade one plugs were placed on the lower right side, grade two plugs were placed on the lower left side, and grade three plugs were placed on the upper left side in a clockwise direction (Figure 1B). Ex vivo osteochondral plug experiments were performed using cartilage obtained from a single donor, and the experiments were independently repeated three times.

### 2.7. Ex Vivo Cell Adhesion Assay

BM-MSCs were carefully detached from the cell culture dishes and thoroughly washed twice with DPBS. Subsequently, the cells were suspended in DMEM-LG at a density of 5 × 10^6^ cells/mL. Then, 1 mM Calcein AM (Sigma-Aldrich) stock was added, mixed carefully, and incubated at 37 °C for 30 min. The cells were then washed twice with DPBS and suspended either in saline (control) or treated with PRP as follows: Control (suspended in saline); PRP (suspended in PRP); pretreated with PRP for 10 min (cells were suspended in PRP for 10 min before the transplant); pretreated with PRP for 30 min (cells were suspended in PRP for 30 min before the transplant). Cells were then added to the culture dish containing the plugs at a concentration of 1.0 × 10^4^ cells/mm^2^ and incubated for 15 min at 37 °C in a humidified 5% CO_2_ incubator. After incubation, the osteochondral plugs were washed four times to remove non-adherent cells and transferred to a 96-well plate with the cartilage surface facing downward. Fluorescent images of cells attached to the osteochondral plug surface were captured using a confocal microscope (Carl Zeiss, Jena, Germany). Fluorescence-labeled cells were quantified using a Multilabel Plate Reader Victor3 (PerkinElmer, Waltham, MA, USA).

### 2.8. Statistical Analysis

Statistical analyses were performed using the IBM SPSS Statistics 26 software (IBM, SPSS Inc., Chicago, IL, USA). All experiments were performed with at least three independent biological replicates, and data are expressed as the mean ± standard deviation. Prior to parametric statistical analysis, normality testing was performed for all in vitro datasets. Because the in vitro data satisfied the assumptions for normal distribution, significant differences among groups were analyzed using a two-tailed Student’s *t*-test and one-way ANOVA. Dunnett’s post hoc test was used for comparisons with the control group, whereas Tukey’s honestly significant difference (HSD) test was used for comparisons among PRP treatment and PRP pretreatment groups for 10 or 30 min. The mean values were considered statistically significant at * *p* < 0.05, ** *p* < 0.01, and *** *p* < 0.001.

For the ex vivo experiments, nonparametric statistical analyses were performed using the Mann–Whitney U test and Kruskal–Wallis test. Values were considered statistically significant at * *p* < 0.05, ** *p* < 0.01.

## 3. Results

### 3.1. PRP Promotes the In Vitro Adhesion and Short-Term Retention of BM-MSCs and Chondrocytes

PRP enhanced the attachment of both BM-MSCs and chondrocytes in a time-dependent manner. Cell attachment was highest in the group pretreated with PRP for 30 min, followed by the 10 min pretreatment group, the PRP-only treatment group, and the control group.

**Figure 2:**
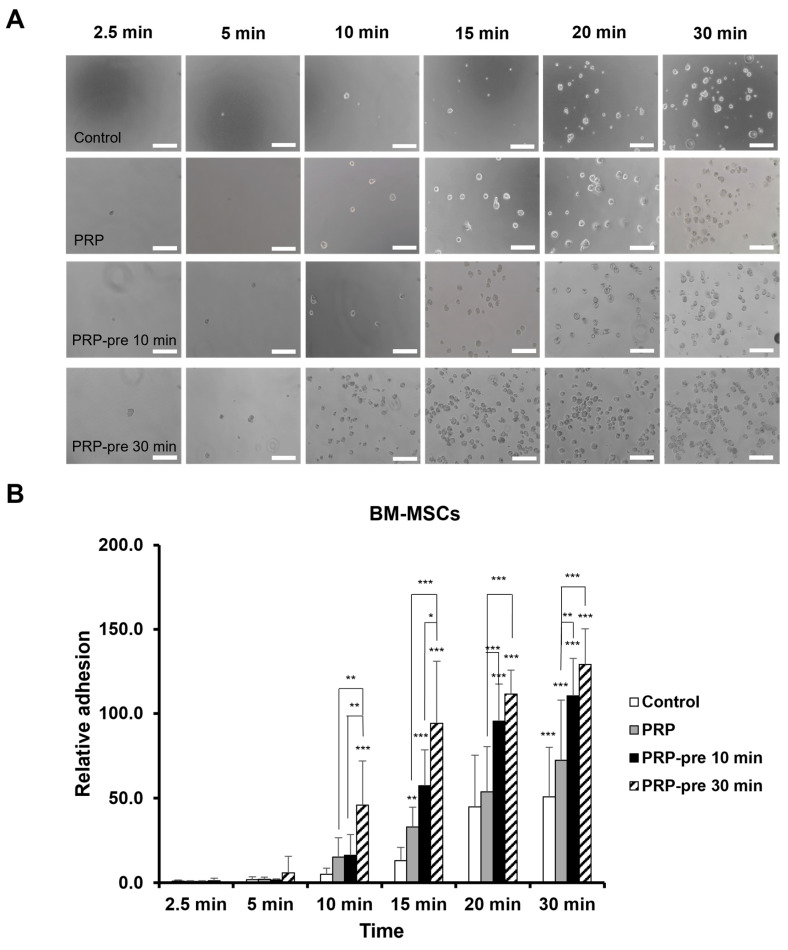
Effects of PRP on BM-MSC adhesion and short-term retention (**A**) Representative phase-contrast microscopy images of BM-MSCs attached to plastic culture dishes (original magnification, 100×; scale bar = 200 µm). (**B**) Quantitative graph showing the effects of PRP on BM-MSC adhesion. Quantitative results are presented as the mean ± SD from three independent biological experiments. The results were analyzed using one-way ANOVA followed by Dunnett’s post hoc test (** *p* < 0.01, *** *p* < 0.001 vs. control; * *p* < 0.05, ** *p* < 0.01, and *** *p* < 0.001 vs. each group).

BM-MSC attachment increased over time and was further augmented by PRP treatment (Figure 2A). After 30 min of incubation, attachment increased by 50.8-fold in the control group, 72.3-fold in the PRP-treated group, and 110.5-fold and 129.1-fold in the groups pretreated with PRP for 10 and 30 min, respectively, compared with the control group at 2.5 min (all *p* < 0.001). No significant difference was observed between the 10 and 30 min pretreatment groups at 20 and 30 min of incubation, suggesting a plateau effect in attachment capacity (Figure 2B).

**Figure 3:**
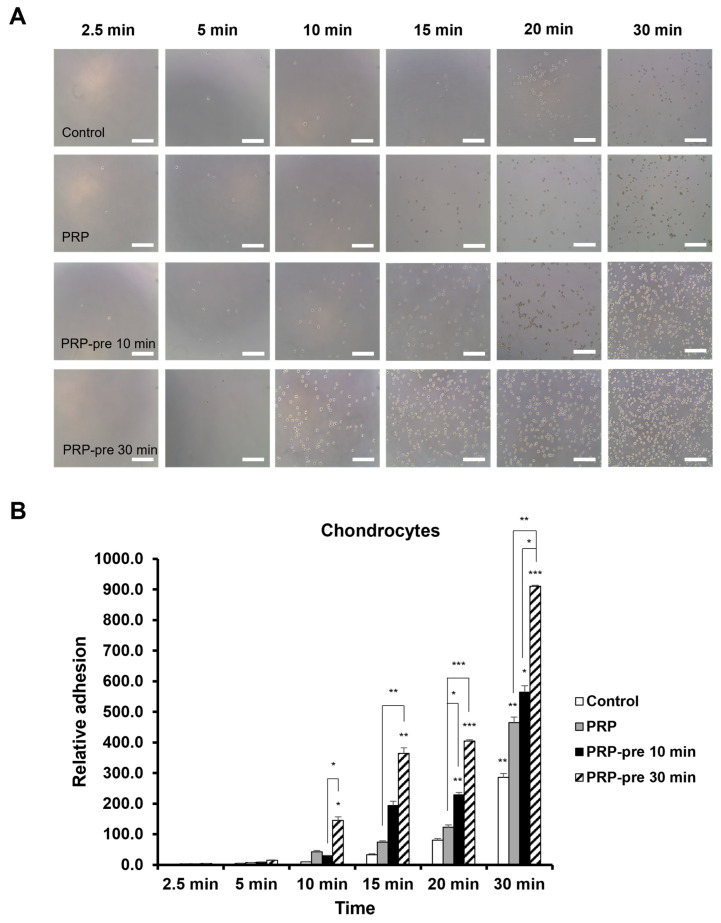
Effects of PRP on chondrocyte adhesion and short-term retention. (**A**) Representative phase-contrast microscopy images of chondrocytes attached to plastic culture dishes (original magnification, 100×; scale bar = 200 µm). (**B**) Quantitative graph showing the effects of PRP on chondrocyte adhesion. Quantitative results are presented as the mean ± SD from three independent biological experiments. The results were analyzed using one-way ANOVA followed by Dunnett’s post hoc test (* *p* < 0.05, ** *p* < 0.01, and *** *p* < 0.001 vs. control; * *p* < 0.05, ** *p* < 0.01, and *** *p* < 0.001 vs. each group).

Chondrocyte attachment also increased in a time-dependent manner and was further enhanced by PRP treatment (Figure 3A). Relative to the control group at 2.5 min, attachment after 30 min increased by 286.3-, 464.8-, 564-, and 910.3-fold in the control, PRP-treated, 10 min pretreatment, and 30 min pretreatment groups, respectively (*p* < 0.01 for all groups except the 30 min pretreatment group, *p* < 0.001). Among the treatment groups, the 30 min pretreatment group showed the highest attachment capacity and remained significantly higher than the 10 min pretreatment group at 30 min of incubation (*p* < 0.05). A plateau in attachment was observed after 30 min of incubation (Figure 3B).

### 3.2. Time-Dependent Effects of PRP Pretreatment on BM-MSC Attachment

Significant differences in BM-MSC attachment among treatment groups became apparent after 10 min of incubation (Figure 2B). The 30 min PRP pretreatment group demonstrated significantly greater attachment than the control and PRP treatment groups at 10 and 15 min of incubation. At 20 and 30 min, both the 10 and 30 min PRP pretreatment groups showed significantly enhanced attachment compared with the control and PRP treatment groups. No significant difference was observed between the 10 and 30 min pretreatment groups at later time points, suggesting a plateau effect in BM-MSC attachment capacity.

### 3.3. Time-Dependent Effects of PRP Pretreatment on Chondrocyte Attachment

Significant differences in chondrocyte attachment among treatment groups became apparent after 10 min of incubation (Figure 3B). The 30 min PRP pretreatment group demonstrated significantly greater attachment than the control group at all later time points and showed the highest attachment capacity overall. At 20 and 30 min of incubation, both the 10 and 30 min PRP pretreatment groups exhibited significantly enhanced attachment compared with the control and PRP treatment groups. Chondrocyte attachment reached a plateau after 30 min of incubation.

### 3.4. PRP Enhances the Adhesion and Short-Term Retention Abilities of Cells Ex Vivo

**Figure 4:**
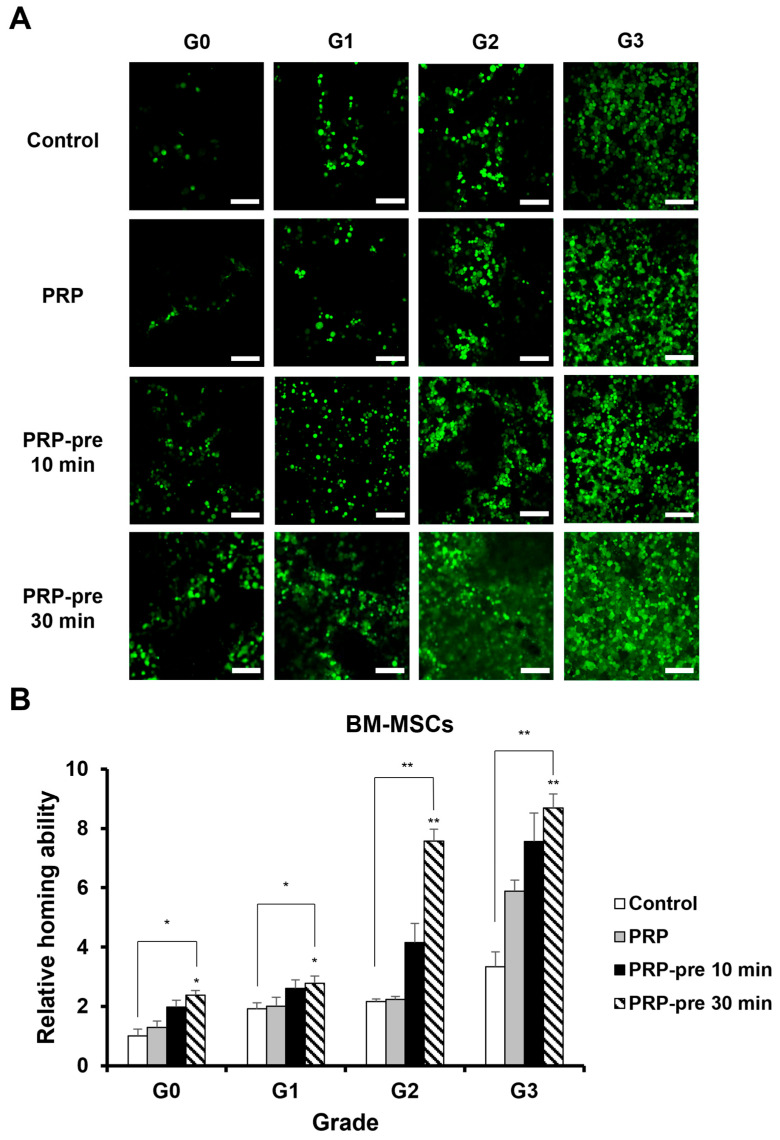
Ex vivo effects of PRP on BM-MSC adhesion and short-term retention (**A**) Representative confocal microscopy images showing the ex vivo effects of PRP on the adhesion and short-term retention of BM-MSCs (original magnification, 100×; scale bar = 200 µm). (**B**) Quantitative graph showing the ex vivo effects of PRP on the adhesion and short-term retention of BM-MSCs. Quantitative results are presented as the mean ± SD from three independent biological experiments. Results were analyzed using the Mann–Whitney *U* test and Kruskal–Wallis test (* *p* < 0.05, ** *p* < 0.01 vs. control).

Representative histological characteristics of articular cartilage according to injury grade are shown in Figure 1A. Representative fluorescent images of BM-MSC attachment to osteochondral plugs in the ex vivo model are shown in Figure 4A.

As the severity of osteochondral plug lesions increased from G1 to G3, the adhesion and short-term retention abilities of the cells increased significantly (*p* < 0.05 and *p* < 0.01, respectively). In line with the in vitro findings, adhesion was highest in the group pretreated with PRP for 30 min, followed by the 10 min pretreatment group, the PRP-treated group, and the control group (Figure 4A,B).

In Grade G2 and G3, adhesion and short-term retention were significantly increased in the groups pretreated with PRP for 30 min compared with the control group (both *p* < 0.01). In Grade G3, these effects were further enhanced, although no significant difference was observed between the 10 and 30 min pretreatment groups. Overall, adhesion and short-term retention increased with lesion severity and were further enhanced by PRP treatment. Additionally, PRP pretreatment for 30 min significantly increased the adhesion and short-term retention capacity of MSCs across all cartilage grades (Figure 4B).

## 4. Discussion

Platelet-rich plasma (PRP) refers to a platelet concentrate with a higher concentration of platelets than that normally found in blood. PRP may contain various components in addition to platelets, including plasma, growth factors, leukocytes, and other blood components. PRP can be derived from autologous or allogeneic sources and can be prepared from either platelets or plasma. Articular cartilage is generally considered an immune-privileged tissue because of its avascular nature and low cellularity, which may partially support the use of allogeneic cell-based therapies in cartilage regeneration. In particular, MSCs exhibit relatively low immunogenicity and possess immunomodulatory properties, making allogeneic MSC applications clinically attractive. However, immune responses may still occur depending on donor–recipient compatibility, inflammatory conditions, and the persistence of engrafted cells. Similarly, PRP products may differ immunologically according to whether they are prepared from autologous or allogeneic sources, as variations in donor-derived bioactive factors and plasma proteins could influence therapeutic responses. In the present study, leukocyte-poor (LP) PRP and BM-MSCs were evaluated under controlled experimental conditions; however, further studies are required to investigate the immunological interactions and therapeutic efficacy of autologous versus allogeneic combinations in vivo and in clinical settings. PRP has received considerable attention for treating musculoskeletal disorders because of its diverse biologically active compounds. A previous study demonstrated that allogeneic pure PRP exerted multiple biological effects on tenocytes depending on the inflammatory state. The treatment did not result in adverse events but instead reduced pain and enhanced shoulder function. The observed improvement was comparable to the effects of steroid injections in patients with rotator cuff tears [32]. Another study indicated that PRP could ameliorate inflammation and promote tissue healing by regulating autophagy and transforming inflammatory molecules into anti-inflammatory molecules [33]. However, despite the promising biological effects of PRP reported in experimental studies, clinical outcomes have not always been consistent across musculoskeletal disorders. For example, systematic reviews and meta-analyses in tendinopathy have reported variable or limited therapeutic benefits of PRP treatment, particularly in Achilles tendinopathy [34,35]. These inconsistent outcomes may be related to differences in PRP composition, platelet concentration, leukocyte content, preparation protocols, and patient-related factors.

The results of the present study demonstrated that PRP enhances the adhesion and short-term retention of BM-MSCs on articular cartilage under ex vivo conditions. The pretreatment duration of 30 min was selected to specifically assess early adhesion-related effects, which occur within minutes to hours following exposure to bioactive stimuli. Longer pretreatment periods may lead to additional biological responses, such as changes in proliferation or differentiation, which were beyond the scope of the present study. These findings suggest that PRP may support early cell attachment processes relevant to stem cell-based approaches; however, their therapeutic relevance requires validation in appropriate in vivo animal models. Because the present study was specifically designed to investigate the early adhesion and short-term retention of MSCs under controlled experimental conditions, in vitro and ex vivo systems were used to minimize the influence of complex intra-articular variables, including synovial fluid composition, inflammatory mediators, and mechanical shear stress. Therefore, additional studies using animal models of cartilage injury or osteoarthritis will be necessary to determine whether PRP-enhanced MSC retention can improve long-term engraftment and cartilage repair under physiological conditions. Previous studies have similarly demonstrated that growth factors in PRP induce chemotactic responses and migration of BM-MSCs in vitro [36,37]. Recent studies have suggested that PRP enhances MSC proliferation, migration, and regenerative activity within degenerative joint environments [38,39,40]. However, relatively few studies have investigated the effects of PRP on the early adhesion and retention of MSCs on damaged articular cartilage. Our group previously reported related intellectual property proposing the use of PRP to enhance the engraftment, attachment, and homing properties of transplanted cells [24]. The present study further extends this concept by experimentally demonstrating that PRP pretreatment enhances the early adhesion and short-term retention of BM-MSCs on damaged articular cartilage under controlled in vitro and ex vivo conditions.

A recent study demonstrated that PRP enhances the proliferation and migration of adipose-derived mesenchymal stromal cells (AD-MSCs), potentially contributing to tissue regeneration [41]. The enhanced adhesion and retention observed in this study may be attributed to several biological mechanisms. Previous studies investigating platelet gel and fibrin gel derived from cord blood demonstrated the presence of regenerative signaling molecules, including TGF-β1, PDGF, VEGF, FGF, ICAM-1, and VCAM-1, which are associated with cell activation, migration, adhesion, and tissue regeneration [42]. Proteomic analyses have further shown that platelet-derived biologics contain diverse signaling molecules, receptors, and adhesion-related proteins involved in wound healing and extracellular matrix (ECM) interactions [42]. These findings suggest that the enhanced short-term retention observed in the present study may not be solely attributable to fibrin formation, but also to bioactive molecules within PRP that modulate early cell–matrix and cell–cartilage interactions. PRP contains high concentrations of chemotactic factors related to stem cell migration and proliferation, including stromal cell-derived factor-1 (SDF-1), platelet-derived growth factor (PDGF), and transforming growth factor-beta (TGF-β) [43,44,45]. These factors may create a bioactive microenvironment that facilitates cell attachment and early retention within the experimental system. The enhanced short-term retention observed in the PRP treatment group may be associated with the anti-inflammatory and angiogenic properties of PRP. The present study did not directly evaluate changes in chemotactic signaling pathways or adhesion-related molecule expression following PRP pretreatment; therefore, the proposed mechanisms remain speculative and require further investigation. Future studies should focus on elucidating the molecular mechanisms underlying PRP-enhanced MSC adhesion and short-term retention. In particular, investigations of adhesion-related molecules, including integrins, ICAM-1, VCAM-1, and focal adhesion-associated signaling pathways, may provide further insight into the mechanisms regulating early cell attachment to damaged cartilage. Furthermore, transcriptomic or proteomic approaches may help identify key bioactive factors within PRP that contribute to enhanced MSC adhesion and retention.

Although articular cartilage lesions are often associated with inflammatory microenvironments [46,47,48], the current study did not directly assess inflammatory modulation. PRP appears to alleviate this inflammatory environment, contributing to the maintenance of BM-MSC multipotency and promoting cartilage differentiation. In the presence of PRP, BM-MSCs showed increased attachment to cartilage explants and maintained chondrocyte-associated phenotypic markers by increasing collagen type II and aggrecan expression. Thus, PRP may facilitate early cell attachment and support phenotypic maintenance under controlled experimental conditions [49].

The limitations of this study are that the results were limited to in vitro and ex vivo models; therefore, further studies are needed to determine whether the same effects are observed in preclinical and clinical settings. In addition, the relatively small sample size and limited number of experimental repeats may restrict the statistical robustness and generalizability of the findings. Furthermore, the ex vivo osteochondral explant experiments were performed using cartilage obtained from a single donor, and donor-specific factors such as cartilage quality, age, and osteoarthritic severity may have influenced the observed cell adhesion and retention results. Therefore, further studies using cartilage samples from multiple donors are needed to confirm the reproducibility and generalizability of the present findings. Although all experiments were performed using three independent biological replicates and demonstrated consistent trends, further studies with larger sample sizes and additional repetitions are warranted to validate the reproducibility of the present findings. Although PRP has shown promising therapeutic potential in musculoskeletal disorders, clinical outcomes have not always been consistent across studies. Variability in platelet concentration, leukocyte content, donor-related factors, and preparation protocols may substantially influence the biological activity and therapeutic efficacy of PRP products. Therefore, caution is required when translating experimental findings into clinical applications, and standardized PRP preparation protocols should be established. Previous studies have emphasized that platelet concentration and contamination with leukocytes or red blood cells may substantially influence the biological activity and regenerative potential of PRP products, highlighting the importance of standardization and characterization of PRP preparations [50]. Future research should investigate the therapeutic potential of combined treatment with PRP and BM-MSCs in appropriate animal models of osteoarthritis and cartilage injury, followed by preclinical and clinical validation studies. Moreover, variables such as the optimal PRP concentration, injection frequency, and injection timing require further investigation. Importantly, the present findings should not be interpreted as direct evidence of in vivo homing or engraftment, as the experimental conditions do not fully replicate the complex intra-articular environment, including synovial fluid composition and mechanical shear stress.

## 5. Conclusions

This study investigated whether platelet-rich plasma (PRP) could enhance the adhesion and short-term retention of bone marrow-derived mesenchymal stromal cells (BM-MSCs) on damaged articular cartilage under in vitro and ex vivo conditions. PRP pretreatment significantly enhanced the adhesion and short-term retention of BM-MSCs on cartilage explants, suggesting that PRP may facilitate early cell attachment processes under controlled experimental conditions. These findings provide additional insight into the potential role of PRP in improving early engraftment-related events in stem cell-based cartilage repair. From a practical perspective, PRP may serve as a supportive biological environment for MSC-based therapies by enhancing early cell retention at cartilage lesion sites following intra-articular administration. Such an approach may help improve the local persistence and therapeutic efficacy of transplanted MSCs in osteoarthritis and cartilage degeneration. Although further studies are required to confirm these effects under in vivo conditions, the combined use of PRP and MSCs may represent a promising strategy for cartilage repair and regenerative treatment.

## Data Availability

The datasets generated and/or analyzed during the current study are available from the corresponding author upon reasonable request.

## Abbreviations

The following abbreviations are used in this manuscript:

MSCs	mesenchymal stromal cells
ATMPs	advanced therapy medicinal products
PRP	Platelet-rich plasma
BM-MSCs	bone marrow-derived MSCs
PSCs	pluripotent stem cells
ASCs	adult stem cells
CSCs	cancer stem cells
DCs	dendritic cells
NK	natural killer
ECM	extracellular matrix
HSC	hematopoietic stem cell
SDF	stromal cell-derived factor
CXCR	CXC chemokine receptor
DMEM-LG	low glucose Dulbecco’s modified Eagle’s medium
PBS	phosphate-buffered saline
ACDA	anticoagulant acid citrate dextrose
BSA	bovine serum albumin
ICRS	International Cartilage Repair Society
